# Intravesical injection of onabotulinumtoxinA in neurogenic overactive bladder patients with human T‐cell leukemia virus type 1‐associated myelopathy: A single‐institution case series

**DOI:** 10.1002/iju5.12301

**Published:** 2021-05-05

**Authors:** Tomohiro Matsuo, Tatsufumi Nakamura, Katsuya Sato, Yasuyoshi Miyata, Hideki Sakai

**Affiliations:** ^1^ Department of Urology Nagasaki University Graduate School of Biomedical Sciences Nagasaki Japan; ^2^ Department of Social Work Faculty of Human and Social Studies Nagasaki International University Sasebo Japan; ^3^ Department of Locomotive Rehabilitation Science Unit of Rehabilitation Sciences Nagasaki University Graduate School of Biomedical Sciences Nagasaki Japan

**Keywords:** human T‐lymphotropic virus 1, intravesical administration, neurogenic urinary bladder, onabotulinum toxin A, tropical spastic paraparesis

## Abstract

**Introduction:**

Neurogenic overactive bladder is a main feature of human T‐cell leukemia virus type 1‐associated myelopathy/tropical spastic paraparesis. We successfully performed intravesical onabotulinumtoxinA therapy for refractory neurogenic overactive bladder due to human T‐cell leukemia virus type 1‐associated myelopathy/tropical spastic paraparesis.

**Case presentation:**

We retrospectively reviewed four neurogenic overactive bladder patients with human T‐cell leukemia virus type 1‐associated myelopathy/tropical spastic paraparesis who underwent bladder wall injections of onabotulinumtoxinA from April to October 2020. All patients were female. Their median age was 66 (range, 63–71) years. They were previously treated with β3‐adrenergic receptor agonists or anticholinergic drugs alone or in combination for ≥12 weeks. However, insufficient results were obtained. After 4 weeks of intravesical onabotulinumtoxinA therapy, overactive bladder symptoms improved and maximum cystometric capacity increased in all cases.

**Conclusion:**

Intravesical onabotulinumtoxinA therapy may be useful for treating refractory overactive bladder due to human T‐cell leukemia virus type 1‐associated myelopathy/tropical spastic paraparesis.

**Abbreviations**
AC: anticholinergic drug
BoNTA: OnabotulinumtoxinA
CISC: clean intermittent self‐catheterization
HAM/TSP: human T‐cell leukemia virus type 1‐associated myelopathy/tropical spastic paraparesis
HTLV‐1: human T‐cell leukemia virus type 1
IPSS: international prostate symptom score
NGB: neurogenic bladder
OAB: overactive bladder
OABSS: overactive bladder symptom scores
OMDS: Osame motor disability score
PdetQmax: detrusor pressure at maximum flow rate
PSL: prednisolone
QoL: quality of life
β3: β3 adrenergic receptor agonist

**Keynote message**

Intravesical onabotulinumtoxinA treatment may be effective in treating refractory neurogenic overactive bladder with HAM/TSP. Since HAM/TSP is a rare disease, large‐scale clinical research is considered difficult to design and carry out. However, prospective studies are needed in future to determine the optimal treatment of this disease.

## Introduction

HTLV‐1 is a retrovirus that has infected approximately 10–20 million people worldwide.^1^, ^2^ High endemic areas include southern Japan, the Caribbean, Central and South America, the Middle East, Melanesia, and the equatorial regions of Africa.^1^, ^2^ HTLV‐1 causes adult T‐cell leukemia and HTLV‐1‐associated myelopathy/tropical spastic paraparesis (HAM/TSP).^3^, ^4^ HAM/TSP is characterized by chronic inflammation of the lower thoracic spinal cord, causing slowly progressing movement disorders of the lower limbs and lower urinary tract symptoms, including OAB.^5^, ^6^

The efficacy of steroids and interferon‐α in the treatment of OAB due to HAM/TSP is limited, and symptomatic treatment plays a central role in the treatment.^6^ Hence, it is crucial that a safer and more reliable treatment strategy to be established for OAB associated with HAM/TSP. In Japan, botulinum toxin intravesical infusion therapy has recently been covered by insurance for patients with refractory neurogenic OAB. Therefore, we report our experience of using botulinum toxin intravesical instillation therapy in patients with neurogenic OAB due to HAM/TSP, whose symptoms did not improve after treatment with conventional medications.

## Case presentation

The protocol for this research project has been approved by the Ethics Committee of the institution (Institutional Review Board of Nagasaki University Hospital, Approval No. 20122138). The study conforms to the provisions of the Declaration of Helsinki. All patients provided written informed consent.

### Patients

The diagnosis of HAM/TSP was established by two neurologists (TN and TS). We retrospectively investigated patients with HAM/TSP whose OAB symptoms did not improve (according to OABSS^7^: urgency ≥2, total score ≥3) after receiving anticholinergic drugs and/or a β3‐adrenergic receptor agonist for ≥3 months.

None of the patients had systemic comorbidities, including neurological disorders associated with OAB other than HAM/TSP.

OnabotulinumtoxinA (200 UI) (BoNTA: BOTOX^®^; GlaxoSmithKline K.K., Tokyo, Japan) was diluted in 30 mL physiological solution. One milliliter of this reconstituted onabotulinumtoxinA was administered by bladder injection needle under cystoscopy (injeTAK®; EDAP TMS Japan, Tokyo, Japan) at 30 different sites of the detrusor muscle under local anesthesia (10 mL of 2% lidocaine). We retrospectively examined changes in subjective and objective symptoms as evaluated by the OABSS, IPSS, and a pressure flow study before and 4 weeks after administration. Table 1 details the patient characteristics before onabotulinumtoxinA treatment.

**Table 1:** Patient characteristics

Case no.	1	2	3	4
Age (years)	66	66	71	63
Gender	Female	Female	Female	Female
Duration of illness (years)				
HAM/TSP	24	12	28	7
NGB	22	9	21	7
OMDS	3	4	4	2
Concomitant therapy				
Immunomodulator	–	PSL	–	–
Drug for OAB	β3	β3+AC	AC	β3
Changes in total OABSS induced by pretreatment of OAB	13 → 11	15 → 14	15 → 15	12 → 8
Duration of pretreatment of OAB (years)	8	9	15	7
Reason for starting BoNTA treatment	Insufficient effect	Insufficient effect	Insufficient effect	Insufficient effect
CISC	Yes	Yes	Yes	No

### Response and patient outcomes

Tables 2 and 3 show changes in subjective and objective symptoms before and after intravesical BoNTA therapy.

**Fig. 1:**
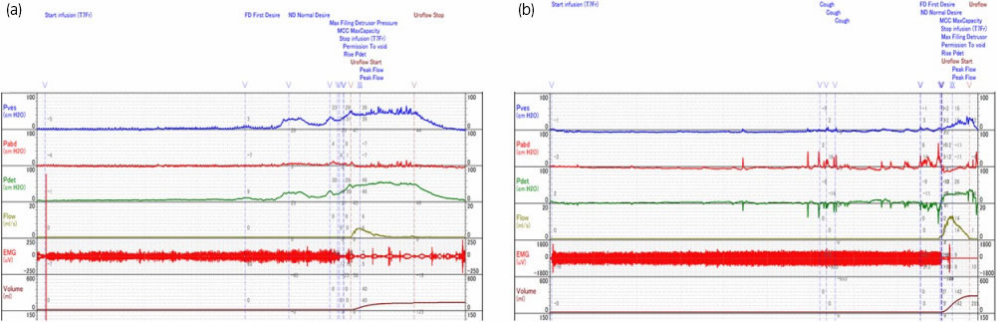
The results of the pressureflow study of case #1 (a, before treatment; b, after treatment). The intravesical onabotulinumtoxinA therapy increases the patient’s maximum cystometric capacity from 268.9 mL to 447.7 mL. The detrusor overactivity (arrow) is confirmed before treatment disappeared after treatment. EMG, electromyography; Pabd, abdominal pressure; Pdet, detrusor pressure; Pves, intravesical pressure.

OABSS improved in all patients, and no patient met the diagnostic criteria for OAB after 4 weeks of BoNTA therapy (Table 2). In addition, all patients showed improvements in total IPSS, IPSS‐storage symptoms, and quality of life scores. However, IPSS‐voiding symptoms worsened in 2 out of 4 cases (50%) (cases # 1 and # 4; Table 2). In the pressure flow study, maximum bladder capacity increased, and detrusor overactivity stopped in all cases. Post‐void residual urine increased in 3 patients (75%; Table 3, Fig. 1).

**Table 2:** Changes in subjective symptoms after treatment

Case No.	1		2		3		4	
	0W	4W	0W	4W	0W	4W	0W	4W
OABSS								
Q1 daytime frequency	1	0	1	0	2	2	1	1
Q2 nocturia	1	0	3	2	3	2	0	0
Q3 urgency	3	1	5	0	5	1	4	1
Q4 urgency incontinence	4	0	5	0	5	1	3	0
Total score	9	1	14	2	15	6	8	2
IPSS								
Q1 incomplete emptying	3	3	2	3	4	4	1	2
Q2 frequency	3	1	4	0	5	2	3	1
Q3 intermittency	2	2	2	2	2	3	0	1
Q4 urgency	4	0	4	0	5	1	4	1
Q5 weak stream	3	3	1	0	3	2	0	0
Q6 straining	3	4	3	2	2	2	1	1
Q7 nocturia	1	0	3	2	3	2	0	0
Storage symptoms (Q2 + Q4 + Q7)	8	1	11	2	13	5	7	2
Voiding symptoms (Q1 + Q3 + Q5 + Q6)	11	12	8	7	11	11	2	4
Total score	19	13	19	9	24	16	9	6
QoL score	5	3	6	2	5	1	4	1

**Table 3:** Changes in objective symptoms after treatment

Case No.	1		2		3		4	
Pressure flow study	0W	4W	0W	4W	0W	4W	0W	4W
First desire to void (mL)	184.8	424.8	137.5	196.5	156.6	243.9	175.5	352.6
Maximum cystometric capacity (mL)	268.9	447.7	139.5	268.4	242.9	352.9	275.2	443.6
PdetQmax (cm H_2_O)	46.5	23.1	25.0	28.4	36.0	28.9	75.5	66.5
Detrusor overactivity	Yes	No	Yes	No	Yes	No	Yes	No
Post‐void residual urine (mL)	148.0	164.0	116.0	110.5	222.0	253.2	88.5	122.1

Case # 1 and case # 3 (2 out of 4) required retreatment 8 and 9 months after the initial BoNTA therapy, respectively.

### Adverse events

None of the patients had serious adverse events such as hematuria, urinary tract infections, or urinary retention.

## Discussion

This was a retrospective study evaluating intravesical BoNTA therapy for patients with OAB due to HAM/TSP. BoNTA, a neurotransmitter blocker, is used to treat OAB and/or neurogenic bladder following detrusor overactivity. In this retrospective analysis, OAB symptoms improved in the patients after treatment.

HAM/TSP is a chronic inflammatory disease of the central nervous system, especially in the lower thoracic spinal cord. Inflammation leads to a slow progression of lower limb sensory disturbance and various lower urinary tract symptoms, including OAB.^5^, ^6^ In addition, the severity of OAB affects patients’ quality of life.^6^, ^8^

The incidence of lower urinary tract symptoms due to HAM/TSP is almost inevitable and considered to be higher than that due to multiple sclerosis (33‐88%).^9^, ^10^ However, it is difficult to distinguish between HAM/TSP and neurogenic bladder due to abnormalities of the central nervous system such as multiple sclerosis using only a urological approach. Therefore, we suggest that close cooperation with neurology specialists is important for diagnosis and treatment.

In general, lower urinary tract symptoms associated with HAM/TSP were initially treated with immunomodulatory therapy such as interferon‐α and corticosteroid hormones, which are therapeutic agents for the primary disease.^6^, ^11^ However, the efficacy of these medications in lower urinary tract symptoms, including OAB, is controversial.^11^ Hence, treatment with β‐3 receptor agonists and anticholinergic drugs plays a central role in OAB with HAM/TSP. However, symptoms may not improve even after treatment with these drugs.

Anticholinergic drugs dampen the amplitude of bladder contractions; improve bladder capacity; and reduce involuntary detrusor contractions, urgency, and frequency.^12^ Anticholinergic drugs, which have been widely recognized as the standard treatment of OAB, are rather difficult to use in patients with HAM/TSP due to their adverse events including dry mouth, voiding difficulty, dry eye, constipation, and cognitive dysfunction, which are autonomic symptoms that might lead to tissue destruction.^6^

β‐3 receptor agent is a specific agonist acting on β3‐adrenoceptors in the human bladder, a stimulation of which leads to active relaxation of the detrusor muscle in the storage phase. This, in turn, increases bladder capacity without exerting an effect on voiding.^13^ However, there is still no evidence concerning the use of mirabegron as a first‐line therapy for neurogenic bladder. ^14^

On the other hand, BoNTA, which is a novel agent for treating OAB including neurogenic pathophysiology, blocks quantal acetylcholine release at the neuromuscular junction. The mechanism of action to treat OAB may also reflect action on afferent pathways.^12^

Previous reports have indicated that BoNTA intravesical instillation therapy was effective in patients with neurogenic OAB caused by multiple sclerosis or spinal cord injury.^15^, ^16^ However, there have been only two reports of the same group on the efficacy of BoNTA in patients with neurogenic OAB due to HAM/TSP refractory to conventional treatment.^17^, ^18^ Carneiro et al. reported that total OABSS improved from 13.0 to 1.0 in 10 patients (3 probable HAM/TSP and 7 HAM/TSP) 30 days after BoNTA treatment.^13^ Objective findings revealed that 66% of patients (2/3) exhibited increased bladder capacity after treatment.^17^ All patients in the two studies had detrusor overactivity before treatment. However, these studies did not examine changes in urodynamic examinations after treatment.

In this study, we recruited 4 patients with OAB due to HAM/TSP. We observed an improvement in subjective symptoms using OABSS, an increase in cystometric capacity. The results of these changes in subjective and objective findings are consistent with other reports by Carneiro et al.^17^, ^18^ Furthermore, we also examined each item of the OABSS and found that the most important symptom of OAB, urinary urgency, improved after BoNTA therapy in all patients. In addition, we found an elimination of detrusor overactivity through urodynamic studies. Post‐void residual urine increased and detrusor pressure decreased slightly in 3 patients (75%). Furthermore, IPSS‐voiding symptoms as subjective symptoms slightly worsened in 50% of patients. We suggest that these results were induced by BoNTA therapy. Therefore, BoNTA therapy exerted a detrusor muscle relaxing effect in patients with OAB due to HAM/TSP.

Three of the 4 participants in this study underwent clean intermittent self‐catheterization prior to treatment and were thought to have originally had detrusor hypoactivity. Fortunately, no patients had urinary retention or urinary tract infections. However, it should be noted that BoNTA therapy may increase the residual urine in these patients. Therefore, stricter follow‐up for increased residual urine and urinary retention is necessary for high‐risk patient categories, such as neurogenic OAB.

This retrospective study confirmed that intravesical BoNTA therapy was effective in patients with HAM/TSP‐induced OAB. In particular, this is the first report of an examination of objective findings using urodynamic studies before and after treatment. On the other hand, the present report has several limitations. The major limitation is the small number of patients included in the study. The observation period was also limited to only 4 weeks. In addition, this study was reviewed retrospectively, and no male patients were included in this analysis. Although it is difficult to carry out large‐scale clinical studies because HAM/TSP is a rare disease, it is important to continue accumulating new detailed cases in future.

## Conflict of interest

The authors declare no conflict of interest.

## Data Availability

Data generated and/or analyzed during this research are available from the corresponding author on reasonable request.
